# A case report: anatomical translocation in tibial insertion of semitendinosus tendon after tibial lengthening

**DOI:** 10.1186/s12891-022-05890-w

**Published:** 2022-10-27

**Authors:** Juncai Xu, Dejie Fu, Yang Peng, Pengfei Yang, Ran Xiong, Kai Lei, Lin Guo

**Affiliations:** Center for Joint Surgery, Southwest Hospital, Army Medical University, Chongqing, 400038 China

**Keywords:** Semitendinosus tendon, Tibial lengthening, Anatomic translocation, ACL reconstruction, Case report

## Abstract

**Background:**

There is the most common method for arthroscopic anterior cruciate ligament (ACL) reconstruction by using the semitendinosus and gracilis tendons and single-tunnel technique. However, anatomic translocation of hamstring tendon attachment is very rare.

**Case presentation:**

A 45-year-old male who need to receive right knee ACL reconstruction due to sprain injury while playing table tennis was found to have a translocation at tibial attachment of semitendinosus tendon. The tibial insertion of left semitendinosus was then explored by ultrasound and found to be identical to the contralateral limb. The patient has a history of bilateral tibial lengthening.

**Conclusions:**

This is the first case as far as we know that reported anatomic translocation of the tibial attachment of the hamstring tendon after tibial lengthening. Surgeons should be aware of this specific situation when hamstring tendon need to be harvested to avoid unnecessary complications.

## Background

Anatomic translocation at hamstring tendon attachment is a rare situation. A firm autograft can be harvested from semitendinosus and gracilis tendons and is widely used in ACL reconstruction with improved outcomes and few complications. The semitendinosus arises from the lower and medial tuberosity of the ischium passing down to form a long tendon ending medially to the proximal tibia. It forms Pes Anserinus (PA) together with tendongs of gracilis and sartorius [1]. Some animal studies have shown that bone lengthening surgery results in a corresponding change in muscle length, but this has not been observed in humans [2–4].

**Fig. 1:**
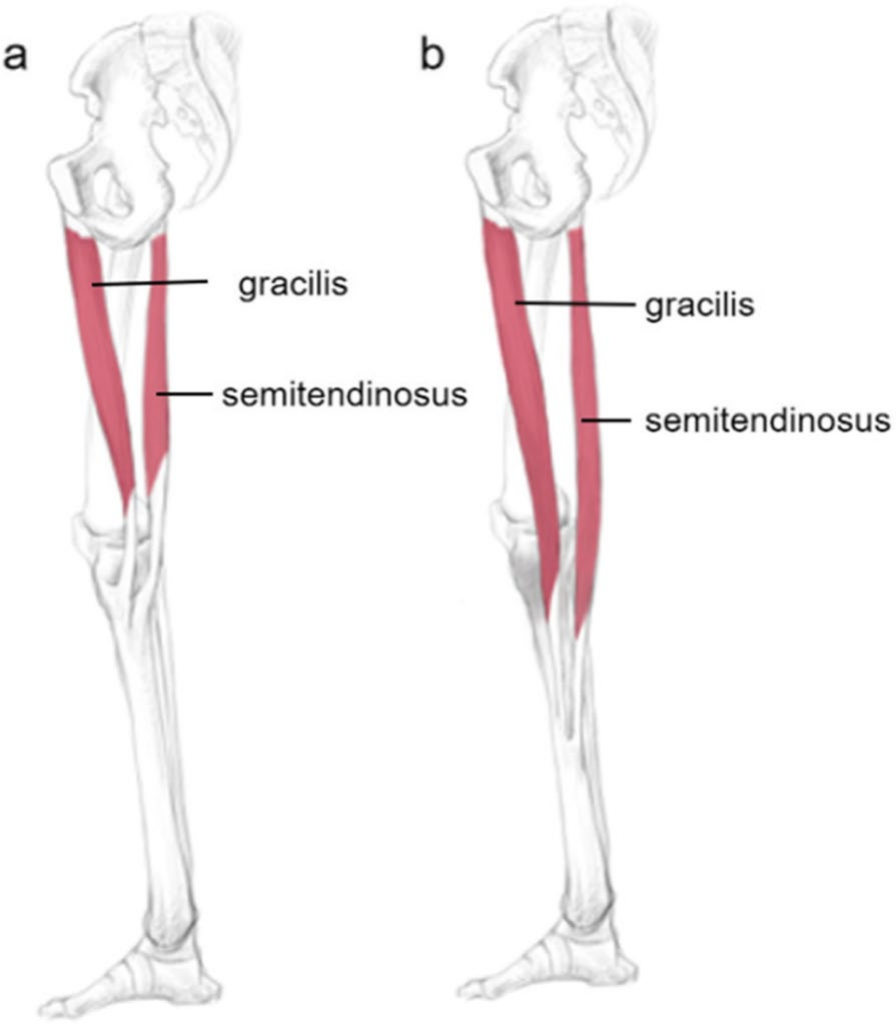
The normal anatomical structure of semitendinosus and gracilis originates from the ischium tuberosity and the pubis and terminates medial to the PA (**a**). The tibial attachment of the semitendinosus and gracilis tendons transferred to the middle tibia after tibial lengthening (**b**)

We present the case of a 45-year-old male patient involving a rare translocation of tibial attachment of the semitendinosus tendon while harvesting hamstring tendons for ACL reconstruction. He had underwent bilateral tibial lengthening 20 years ago (Fig. 1).

## Case presentation

A 45-year-old male referred to our institution who had a twisting injury to his right knee with a popping sensation while playing table tennis 11 months ago, causing joint pain and swelling. The patient was able to walk normally after rest and relaxation but complained of low pain and recurrent joint instability during running and other low-energy activities. He was diagnosed of right ACL rupture and tear of the posterior corner of the medial meniscus by Lachman, pivot shift tests and magnetic resonance imaging (MRI) (Sonata Magnetom, Siemens Medical Solutions, Erlangen, Germany). After a discussion of his options, the patient elected for an ACL reconstruction under arthroscopy using a autograft hamstring tendon.

The patient was otherwise healthy besides undergoing tibial lengthening 20 years ago. Bilateral tibia were lengthened by 5 cm with the osteotomy just superior to pes anserinus. At present, the patient is 170 cm tall and weighs 65 kg. Two months after surgery, the patient was walking normally without crutches and gradually returning to sport. He returned to normal athletic sports two years after surgery with no particular discomfort.

**Fig. 2:**
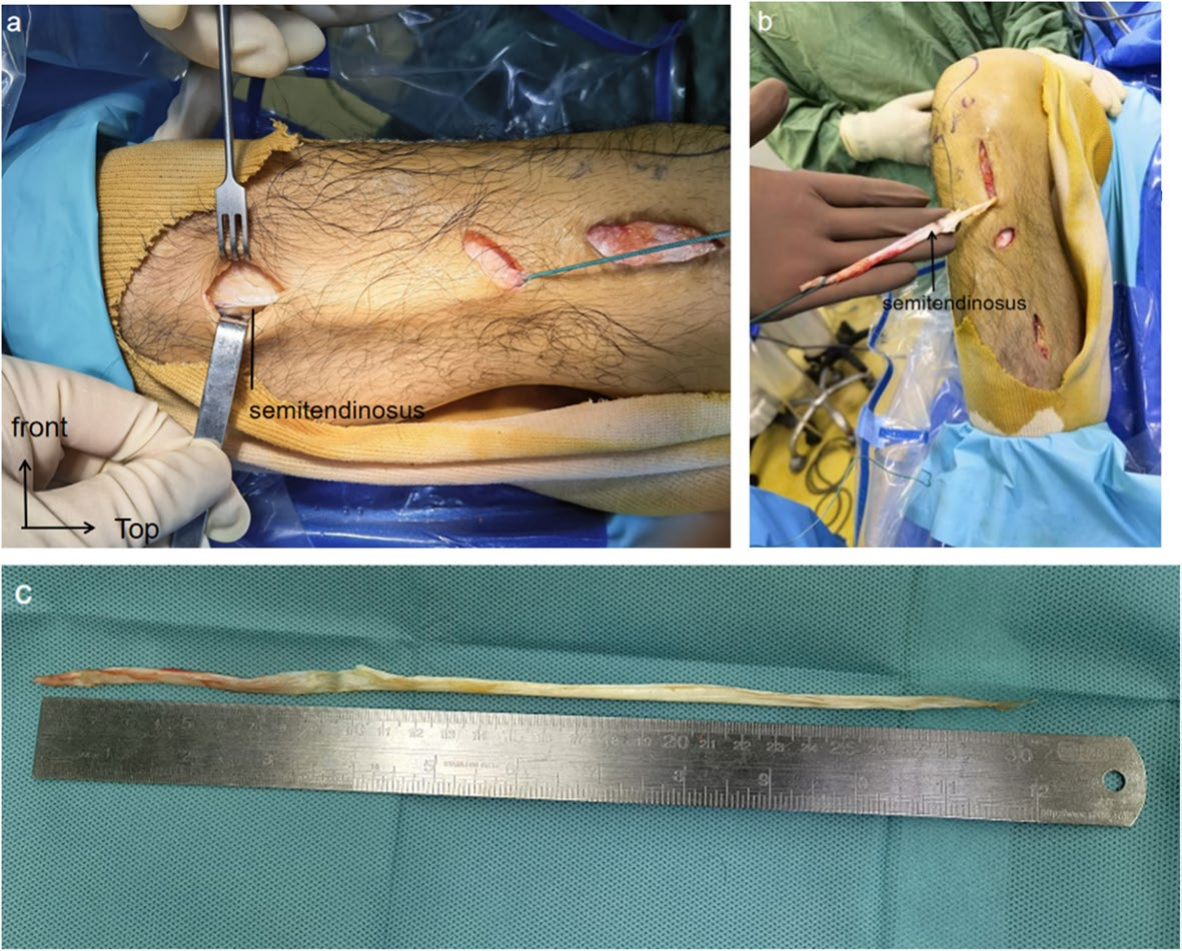
The tibial attachment of the left semitendinosus tendon was found to be obviously abnormal during ACL reconstruction, which was located in the middle of the tibia (**a**). After the tibial attachment of semitendinosus tendon was detached, the end of the tendon was about 13 cm from the PA (**b**). The tendon length of 30 cm is within the normal range (**c**)

The patient was positioned supine and a tourniquet was inflated to 280 mmHg on his right thigh. It was planned to harvest hamstring tendon via the longitudinal 3-cm original surgical incision medial to tibial tubercle. After dissection of subcutaneous tissue, we found that the distal insertion of gracilis and semitendinosus tendon was not located at the common PA postition. The gracilis tendon is located medial and lower to the incision and is 20 cm in length. And the semitendinosus tendon crosses over the medial collateral ligament and extends distally so that we had to make two incisions at the distal tibia to search its insertion. Ultimately we found that semitendinosus tendon was 30 cm in length and the insertion site was 13 cm below the common position and 16 cm from the midpoint of the tibial tuberosity (Fig. 2). Then, the 5-strand graft (3-strand semitendinosus and 2-strand gracilis tendon) with a diameter of 8 mm and length of 8 cm was prepared for ACL reconstruction using a fixed-loop device in the femoral fixation and bioabsorbable interference screw in the tibial fixation.

**Fig. 3:**
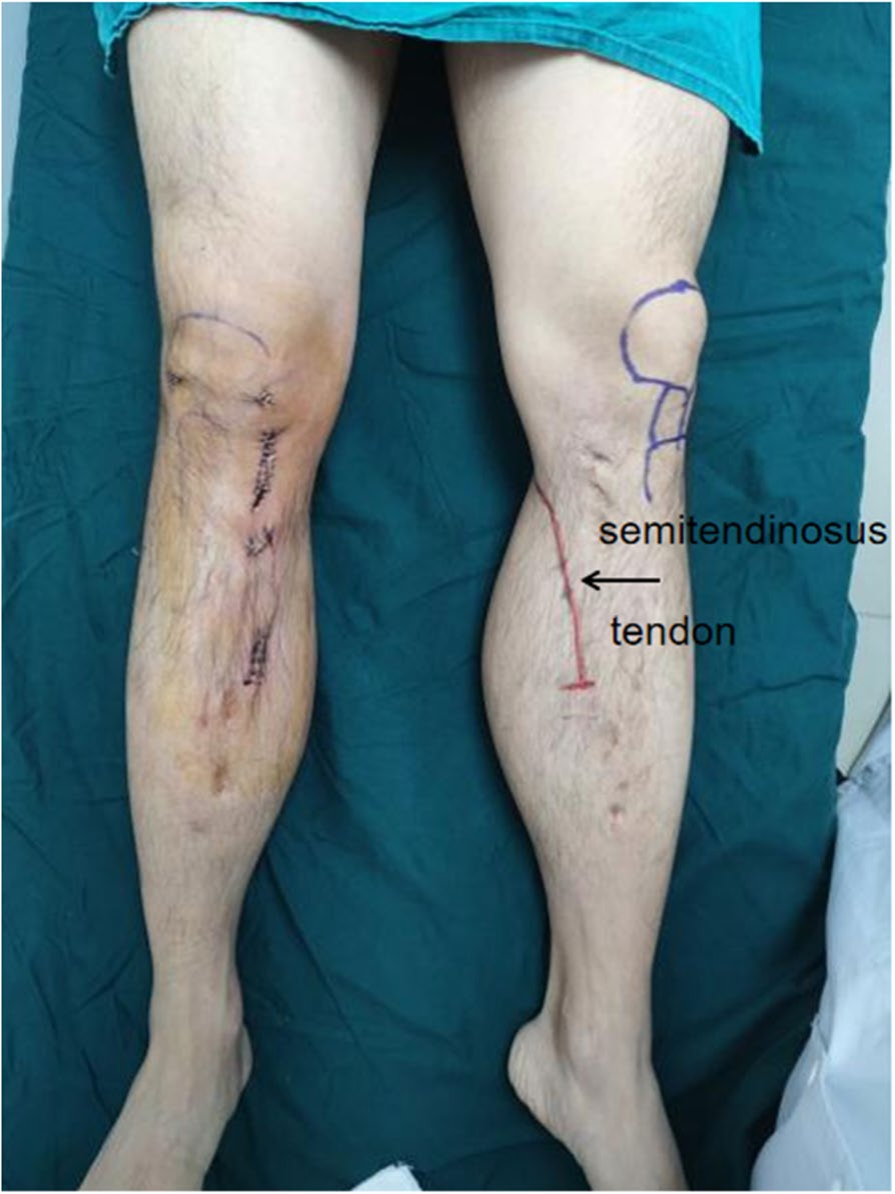
The red line shows the tibial attachment of the left semitendinosus tendon

**Fig. 4:**
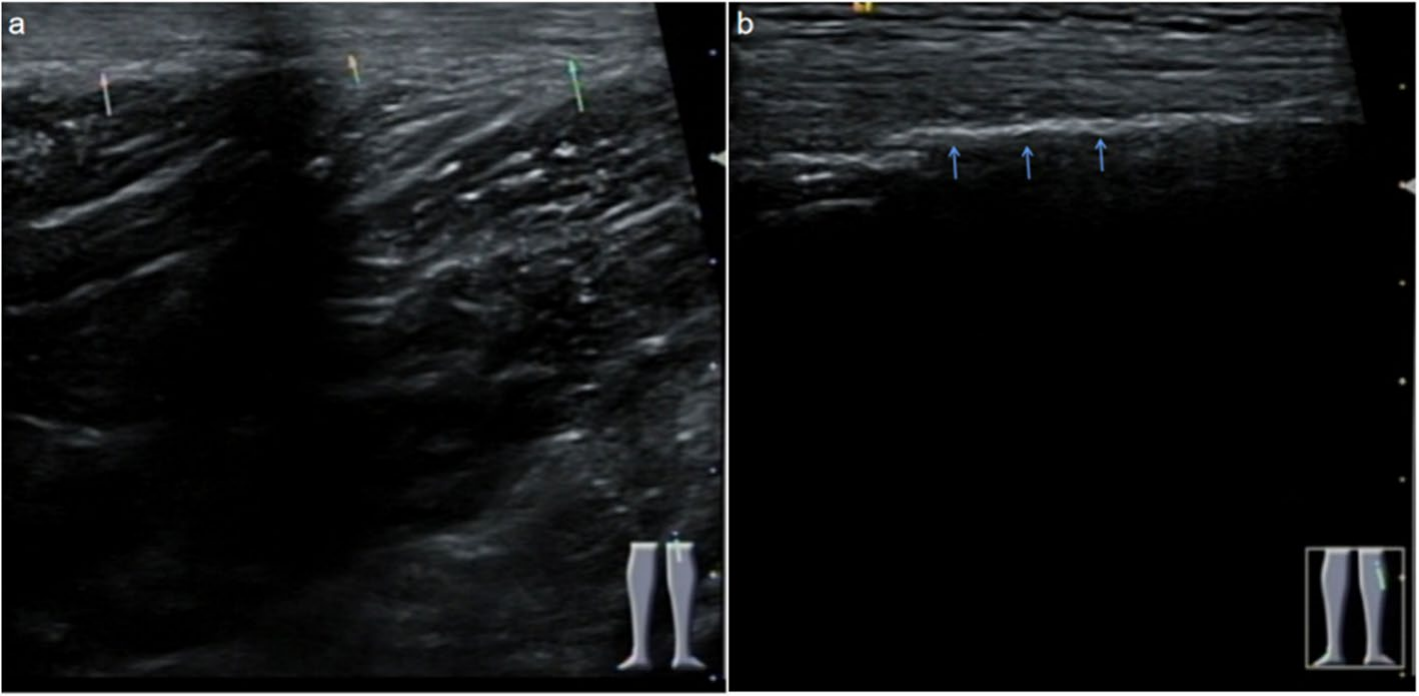
Ultrasound was used to explore the left semitendinosus tendon at the position of the popliteal fossa (**a**) and tibial attachment (**b**)

Ultrasound and MRI were used to explore the contralateral semitendinosus tendon with the patient's consent (Fig. 3) but the path of tendon was found only in ultrasound. We found that the left semitendinosus tendon crosses the popliteal fossa and passes distally from the medial side of the original incision and terminates in the middle of the tibia as does the right (Fig. 4).

The patient performed a specific postoperative rehabilitation training, which consisted of icing, straight leg raise, passive and then active mobilization to obtain a normal range of motion and muscle strengthening (especially quadriceps, hamstrings). The patient achieved full normal range of motion in knee flexion and extension and walked normally without crutches at 6 weeks after surgery. He were able to do low-energy exercise 3 months after surgery such as swimming, jogging and bicycling (indoor) etc. The patient was free to do non-confrontational exercise without any discomfort at six months postoperatively. The quadriceps and hamstring muscle strength of the operative limb is almost the same as that of the contralateral limb. There were no postoperative adverse events.

## Discussion and conclusions

To the best of our knowledge, this is the first report of the translocation in anatomy hamstring insertion after tibial lengthening. Our case implicates that limb lengthening effected not only length of the bone but also effected the insertion of tendon unevenly.

Limb lengthening has long been a popular method for reconstructing or correcting shortening deformity of lower extremity caused by developmental and post-traumatic reasons or tumor surgery, et al. [5–7]. Several studies suggested that distraction osteogenesis enhances the regeneration of all limb tissues including bone, muscle, vessels and skin [8, 9]. Muscles are known to be highly adaptable to mechanical requirements, so the surrounding muscles and tendons grow in parallel direction with the stretched bone during treatment [10].

During tibial lengthening, distraction osteogenesis leads to increased skeletal muscle tension, which is driven by the passively mechanical stretch. Muscle contraction is generated by sarcomeres, which are the minimum functional units of muscle. To maintain muscle contraction forces, muscles are thought to increase the number of sarcomeres in each muscle fiber by adding new sarcomeres in series to reduce sarcomere length [2–4]. With the increase of sarcomeres, muscle metabolism also also greatly accelerated during tibial lengthening [11, 12].

In addition, a study had shown that only muscle contributed to the adaptation to the stretch in adult animals, while both tendon and muscle adaptation were investigated in immature animals [13]. In the present study, this conclusion was indirectly supported by the fact that the length of the semitendinosus tendon obtained during surgery was normal.

The increase of sarcomere and the acceleration of metabolism cannot well explain the phenomenon of the variation in anatomy semitendinosus tendon insertion. There is no literature on tendon insertion translocation after limb lengthening, the reason for which should be clarified in future studies.

In conclusion, this is the first case as far as we know that reported anatomic translocation of the tibial attachment of the hamstring tendon after tibial lengthening. Surgeons should be aware of this specific patient with a history of tibial lengthening when hamstring tendon need to be harvested. It is recommended to perfect preoperative imaging to more accurately explore the attachment point and integrity of the hamstring tendon, which could reduce operation time and avoid excessive surgical incisions like in our case.

## Data Availability

Not applicable.

## Abbreviations

ACL: Anterior cruciate ligament
PA: Pes Anserinus
MEI: Magnetic resonance imaging
